# Epidemiological Associations between Rabies Vaccination and Dog Owner Characteristics

**DOI:** 10.3390/vaccines11020352

**Published:** 2023-02-03

**Authors:** Yuri Amemiya, Satoshi Inoue, Ken Maeda, Hiroshi Nishiura

**Affiliations:** 1 School of Public Health, Kyoto University, Yoshida-Konoe-cho, Sakyo-ku, Kyoto 606-8501, Japan; 2 Department of Veterinary Science, National Institute of Infectious Diseases, 1-23-1 Toyama, Shinjuku, Tokyo 162-8640, Japan

**Keywords:** rabies, vaccination, dog owner, dog registration, association

## Abstract

Background: The annual rabies vaccination coverage in dogs among 47 prefectures in Japan has been reported to range from 42.3% to 92.4%, and the overall coverage has been steadily declining. Given the presence of unregistered dogs and the small number of stray dogs, the true vaccination coverage is likely to be even lower. Methods: We conducted a cross-sectional survey of dog owners to identify the owner characteristics associated with dog rabies vaccination. People in Japan who currently own dogs were recruited and answered a questionnaire consisting of four sections: (i) demographic characteristics, (ii) education history associated with medicine, (iii) factors related to veterinary services, and (iv) dog characteristics. Results: A total of 534 dog owners covering 629 dogs were surveyed. Vaccination within the prior 12 months was the major outcome (56.1%). The associated variables were (a) owner education level, (b) knowledge about mandatory vaccination, (c) having a family veterinary clinic, (d) frequency of visiting a veterinary clinic, and (e) having ever been advised to vaccinate their dog. Conclusions: Although causality cannot be implied, our findings indicate improving owners’ knowledge about mandatory vaccination, facilitating attachment to a veterinary clinic, and veterinarians providing vaccination advice might increase the uptake of dog rabies vaccination. The finding in Japan did not deviate from Asian and African countries with rabies, and the sample estimate of annual vaccination coverage was lower than the reported estimate among registered dogs.

## 1. Introduction

Rabies is a largely neglected tropical disease and is a neuro-invasive zoonosis caused by lyssavirus of the Rhabdoviridae family and the World Health Organization aims to eliminate rabies transmitted from dogs by 2030 [1]. Rabies is primarily transmitted through saliva after a person or animal has been bitten by a rabies-infected animal, such as a dog, fox, raccoon, mongoose, or ferret badger [1]. Dogs are the most common source of rabies transmission to humans, and 99% of rabies deaths in humans originate from a dog bite [2]. Rabies infection in animals is seen in Asia, Africa, North America, and Latin America, but the frequency of infection event is higher in Asia and Africa than other places, particularly in low-income countries located in sub-Saharan Africa [3]. Vaccination in dogs would be an effective countermeasure to prevent rabies, and the World Health Organization recommends achieving a goal of 70% vaccination coverage in dogs [4].

Japan has been free from rabies in domestic dogs and humans for 66 years. The last cases of rabies in humans were reported in 1956, except for four imported cases in 1970, 2006, and 2020 [5,6]. In Japan, dog owners are required to register their dogs with the local government during the dog’s first year of life; additionally, the rabies prevention law states that dog owners must have their dogs vaccinated for rabies annually [7,8]. The local governments then, in turn, issue registration and vaccination tags [8]. To promote vaccination in dogs, local governments conduct mass rabies vaccination programs from April to June every year. The Japanese Ministry of Health Labour and Welfare (MHLW) estimates the annual rabies vaccination coverage in dogs, which was 70.2% in 2020, with coverage decreasing every year since 1996 [7]. However, it is difficult to know the exact rabies vaccination coverage in dogs in Japan because some dogs are unregistered, and there is also a small population of stray dogs. The actual risk of introducing rabies into the dog population and subsequent spreading is therefore largely unknown.

The characteristics of dog owners that are associated with rabies vaccination in dogs have been previously investigated, mainly in African countries. Regarding owner demographics, the following characteristics have been reported as positively associated with dog rabies vaccination: higher education level [9,10,11,12,13], older age [12,13], higher socioeconomic level [12,14], fewer people living in a household [10,15], having an occupation [9,10,12,13], being part of a religious group (Muslim) [9], living in an urban area [9,10], and having a private residence [11]. Other factors include knowledge about rabies [9,11,12,14,16], knowledge about the location of veterinary offices/clinics [11], the breed of dog (exotic/hybrid vs. local) [11,12,14], where the dog was obtained (purchased vs. given) [10,12,14], confinement status of the dog home (i.e., confined vs. free-roaming) [12,14], perception of the rabies vaccination cost [12], and providing veterinary care to owned dogs [10]. It has been documented that rabies-related knowledge, attitudes, and practices are positively associated with the owner’s education level [17,18,19]. A study in China found that owners not recognizing the need for vaccination or not being aware of local policies about rabies vaccination were negatively associated with vaccination in dogs [20]. Studies in Thailand and Laos People’s Democratic Republic suggested that lower education level of owners and having a free-roaming dog were negatively associated with vaccinating dogs [21,22]. According to a study from Haiti, higher willingness to pay for rabies vaccination was negatively associated with vaccinating dogs. The willingness to pay for a dog’s rabies vaccination was higher among people who owned dogs for the purpose of companionship, those who had free-roaming dogs, and those who owned dogs that were unable to be walked with a leash [23].

Little is known about rabies vaccination coverage and its determinants in East Asia, especially in high-income countries such as South Korea, Taiwan, and Japan. Understanding the causal factors associated with rabies vaccination in dogs and the differences in those factors between East Asian and African countries is vital for developing future interventions to increase dog rabies vaccination coverage. The purpose of the present study was to identify associations between rabies vaccination and dog owner characteristics in Japan by using a cross-sectional epidemiological survey.

## 2. Materials and Methods

### 2.1. Epidemiological Data

A cross-sectional survey of people who currently owned dogs in Japan was conducted between 31 August and 7 September 2022. The study participants were non-randomly sampled from a list of registered users of a Japanese internet research company called Mellinks Ltd. [24]. Manual recruitment was conducted by area sampling to obtain participants with age and residential area profiles that were proportional fractions of Japan as a whole. First, a pre-questionnaire survey was conducted to screen eligible participants to identify whether the responder owned one or more dogs. The main investigation survey was then conducted with people who currently owned at least one dog. The survey consisted of four sections, namely, (i) owner demographic characteristics (age, sex, number of household members, residential type (a private house, condominium, or apartment), education level, household income), (ii) education history associated with medicine (being in an occupation associated with human or animal health, knowledge about mandatory dog rabies vaccination, mass vaccination opportunity, and owners’ vaccination history), (iii) variables associated with veterinary service (having a family veterinary clinic and frequency of visiting veterinary clinics), and (iv) dog characteristics (the owners able to interact with other owners, how the dog was obtained (purchased vs. brought/given), where the owners keep their dog (outdoor vs indoor), the age of the dog, vaccination history for rabies and other diseases, and whether the dog is registered or not). As for education history of owners associated with medicine, we assumed that the owners’ own vaccination history (e.g., against COVID-19 or influenza) is associated with vaccine acceptance in the owner and also influences the decision to vaccinate their dogs. The survey was conducted in Japanese; an English translation of the questionnaire is available in the Supplementary Materials.

### 2.2. Data Analysis

The primary outcome was rabies vaccination in dogs within the previous 12 months, treated as a dichotomous variable. The secondary outcomes were dog registration with the local municipality and owners vaccinating their dogs for diseases other than rabies. Among the surveyed items, household income, number of household members, education history, influenza vaccination history, interaction with other dog owners, and frequency of visiting veterinary clinics were treated as ordinal variables; all other variables were treated as dichotomous variables.

Statistical associations between the outcomes and surveyed items were analyzed using univariate Fisher’s exact test or the χ^2^-test, and we then calculated the odds ratios (ORs) of the outcomes (e.g., OR of rabies vaccination). The level of significance was set at α = 0.05. The 95% confidence intervals (CIs) were calculated using the Agresti score confidence interval method.

As an alternative outcome, we examined the total number of rabies vaccinations in a dog divided by the dog’s age. Differences in means were analyzed using Welch’s analysis of variance (ANOVA) and Student’s *t*-test. All statistical data were analyzed using JMP statistical software, version 16.0 (SAS Institute Inc., Cary, NC, USA).

### 2.3. Ethical Considerations

This study was approved by the Medical Ethics Board of the Graduate School of Medicine at Kyoto University (R3660). The study participants, dog owners, were asked to review the consent document prior to answering the questionnaire survey, and only those who agreed on the webpage were invited to complete the questionnaire. After the internet survey was completed, Mellinks compiled anonymously processed information that could not be linked to any personally identifying information.

## 3. Results

A total of 534 dog owners and 629 dogs were enrolled in the study. Table 1 presents the overall characteristics of the participants. The rabies vaccination coverage in dogs within the previous 12 months was estimated at 56.1% (95% CI: 50.9, 61.3). Regarding secondary outcomes, 89.3% (95% CI: 86.8, 91.9) of the dogs were registered, and 81.7% (95% CI: 78.4, 85.1) had received at least one vaccination other than rabies. Associations among outcome variables were evident, e.g., registration (OR = 54.1, *p* < 0.001) and vaccination for diseases other than rabies (OR = 8.2, *p* < 0.001) were positively associated with dogs having had their rabies vaccination. The owners who vaccinated their dog for diseases other than rabies were more likely to register their dog with the local municipality (OR = 5.3, *p* < 0.001).

The mean (standard deviation) of the ratio of the total number of rabies vaccinations per year to dog age was 0.66 (0.48). Employing Welch’s ANOVA, the mean of the ratio was found to be different when grouping the dogs according to the following owner characteristics: (i) knowing that vaccination was mandatory by law (*p* = 0.012), (ii) knowing that mass vaccinations took place once a year (*p* < 0.001), (iii) having a family veterinary clinic (*p* = 0.007), and (iv) the frequency of visiting a veterinary clinic (*p* = 0.005).

Table 2 summarizes the univariate associations between dog rabies vaccination (i.e., vaccinated within the past 12 months) and owner characteristics. Among the owner demographic variables, those who had a university education were more likely to vaccinate their dog against rabies than those who had only junior high school education (OR = 2.5, *p* = 0.014). Regarding the owners’ education about human and animal health, knowing that rabies vaccination was mandatory by law (OR = 2.9, *p* < 0.001) and knowing about the mass vaccination opportunities that occur every year (OR = 2.9, *p* < 0.001) were associated with dog rabies vaccination. The owners who had a family veterinary clinic (OR = 2.3, *p* < 0.001), visited an animal clinic more than once every 6 months (OR = 2.0, *p* = 0.011), visited an animal clinic more than once a month (OR = 2.0, *p* = 0.009), and those who had ever been advised to vaccinate their dog (OR = 2.6, *p* = 0.019) were more likely to vaccinate their dog against rabies. We did not identify any dog characteristics that were significantly associated with dog rabies vaccination.

Regarding demographic characteristics, geographic area of residence (western or eastern Japan) was not associated with dog rabies vaccination (OR = 0.8; 95% CI: 0.6, 1.0; *p* = 0.091). Regarding dog characteristics, the number of dogs per owner was not associated with dog rabies vaccination (OR = 1.2; 95% CI: 0.9, 1.7; *p* = 0.286).

Table 3 summarizes the owner characteristics associated with non-rabies vaccinations. Among the demographic variables, the owners who lived in urban areas were more likely to vaccinate their dogs against diseases other than rabies (OR = 1.8, *p* = 0.005). Regarding the owners’ education about human and animal health, having an education history associated with medicine (OR = 2.3, *p* = 0.005), having an occupation related to dogs (OR = 3.9, *p* = 0.014), having a COVID-19 vaccination history (OR = 2.4, *p* = 0.001), having a yearly influenza vaccination (OR = 1.8, *p* = 0.027), and being willing to receive an influenza vaccination in the future (OR = 1.7, *p* = 0.017) were associated with vaccinating dogs for diseases other than rabies. The owners who had a family veterinary clinic (OR = 3.7, *p* < 0.001), visited an animal clinic more than once every 6 months (OR = 2.1, *p* = 0.010), and visited an animal clinic more than once a month (OR = 2.4, *p* = 0.005) were more likely to vaccinate their dog for diseases other than rabies. Among the dog characteristics, owners interacting with other dog owners once per week (OR = 1.9, *p* = 0.008), owners’ experience with the dog community (OR = 2.2, *p* = 0.014), owners allowing their dog to live indoors (OR = 3.1, *p* < 0.001), and owners’ perception of their dogs as members of the family (OR = 2.7, *p* = 0.017) were positively associated with vaccinating their dogs for diseases other than rabies.

Table 4 shows the univariate associations between owner characteristics and dog registration with the local municipality. Among the demographic variables, owners who thought that it was inexpensive to vaccinate their dog were more likely to register their dog with the local authorities (OR = 1.9, *p* = 0.013). Regarding the owners’ understanding of medicine and animal health, knowing that dog rabies vaccination was mandatory by law (OR = 5.9, *p* < 0.001), knowing that mass vaccination opportunities occurred every year (OR = 3.2, *p* < 0.001), a belief that the COVID-19 vaccine should be taken even if there is a cost for it (OR = 1.9, *p* = 0.025), and receiving a yearly influenza vaccination (OR = 1.9, *p* = 0.047) were positively associated with dog registration. The owners who had a family veterinary clinic (OR = 3.9, *p* < 0.001) visited an animal clinic more than once every 6 months (OR = 5.1, *p* < 0.001), visited an animal clinic more than once a month (OR = 6.4, *p* < 0.001), and those who had ever been advised to vaccinate their dog (OR = 2.4, *p* = 0.004) were more likely to register their dog. Regarding the dog characteristics, interacting with other dog owners at least once every 6 months (OR = 2.8, *p* = 0.011), interacting with other dogs at least once a week (OR = 2.8, *p* = 0.003), and having experience with a dog community (OR = 2.8, *p* = 0.013) were positively associated with dog registration.

## 4. Discussion

The present study investigated statistical associations between rabies vaccination in dogs and dog owner characteristics in Japan. Owners whose highest education level was university or graduate school were more likely to vaccinate their dog than people with a lower education background. Similarly, owners who had a family veterinary clinic, visited a veterinary clinic at least once every 6 months, and who had ever been advised to vaccinate their dog against rabies were more likely to vaccinate their dog against rabies. The identified factors were broadly consistent with findings about dog owner characteristics in other country settings [9,10,11,12,13,17,18,19]. While the total number of dogs is not available and thus it has been difficult to estimate the annual rabies vaccination coverage in dogs, we successfully obtained a sample estimate of the annual vaccination coverage against rabies in dogs and identified differences in the characteristics of dogs related to vaccination by the number of rabies vaccinations.

To the best of our knowledge, the present study is the first in Japan to identify dog owner characteristics that are significantly associated with annual dog vaccination against rabies. The finding in Japan did not deviate from Asian and African countries with rabies. Moreover, although our cross-sectional study cannot demonstrate causal links, the findings imply that improving knowledge about the mandatory annual vaccination and about the opportunity for mass vaccination events may contribute to increasing rabies vaccination in dogs. In African and Asian studies, one of the reasons why owners did not participate in rabies mass vaccination campaigns was insufficient information about the vaccination campaign [25,26,27]. Similarly, increased interactions with veterinarians, including advice to vaccinate one’s dog, may increase vaccination coverage. In the present study, the number of household members, household income, and perception of vaccination cost were not significantly associated with rabies vaccination; however, these factors were significantly associated in studies conducted in Africa [10,12,14,15]. In African countries, there is a wide gap in the post-graduate learning opportunity between those who are rich and those who are poor [28]. Furthermore, this gap indicates a strong positive correlation between the school enrollment rate and socioeconomic factors such as education levels and income [28]. Moreover, the secondary school enrollment rate is about 45% in Sub-Saharan Africa [29]. In contrast, in Japan, the current enrollment rate of upper secondary school including high school has reached 98%, and the university enrollment rate in Japan is 51% [30]. The gap between the rich and the poor seems to be smaller than in African countries; furthermore, the sense of danger regarding rabies in Japan is low due to its highly sanitary environment. Therefore, it is thought that the survey results were not a reflection of an income gap. The associations with the socioeconomic aspects may depend on the context within a country, such as its socio-cultural and socioeconomic backgrounds and geographic characteristics. For instance, in Japan, a small proportion of people have dogs for the purpose of guarding, while a large proportion of owners perceive their dogs to be companion animals. The owners of dogs with a higher ratio of total rabies vaccinations to dog age were more likely to know about mandatory vaccination and mass vaccination opportunities and have a family veterinary clinic and visit a veterinary clinic at least once every 6 months. These findings were consistent with the result using the dichotomous outcome of vaccination within the past 12 months.

We also identified owner characteristics that were associated with dog registration and vaccination for diseases other than rabies. The registration outcome assessed was whether the dog had ever been registered, and the other vaccination outcome was whether the dog had ever received a non-rabies vaccination. Therefore, the number of associated variables was more numerous than those used when analyzing rabies vaccination. We found that owners who had an education history of medicine, pharmacology, or veterinary medicine had an occupation related to dogs, or who recognized that vaccination is effective against human infections were more likely to vaccinate their dog for diseases other than rabies. The results suggested that owners who undergo vaccinations themselves may also be more likely to vaccinate their dog. In Japan, vaccinating a dog for diseases other than rabies (i.e., combination vaccines) is sometimes required for obtaining dog insurance and using dog parks, grooming shops, hotels, and cafes that allow dogs. Many grooming shops and cafes that allow owners to bring their dog are located in urban areas, and experience with participating in a dog community, frequently interacting with other owners, owners’ perception of the dog as a member of the family, and living in urban areas were associated with non-rabies vaccination in dogs.

The positive associations between dog registration and rabies and non-rabies vaccination imply that the owner characteristics associated with dog registration are similar to those associated with rabies and non-rabies vaccination. In municipalities, dog registration and rabies vaccination are often carried out at the same time, e.g., as a combined service at a veterinary clinic when the owner first obtains the dog, when a dog is a puppy, or when the owner moves and changes address. Thus, even though the survey question was focused on rabies in its context, an owner’s understanding of the rabies vaccination policy and vaccination opportunities and being advised by a veterinarian about rabies vaccination were positively associated with dog registration. Dog registration was positively associated with vaccination for other diseases, and the owner’s personal vaccine acceptance and their relationships with veterinarians and other dog owners were found to be positively associated with dog registration.

Rabies vaccination coverage in dogs within the past 12 months was lower than the coverage reported by MHLW [7]; thus, many dogs are not continuously followed up in Japan. The number of dogs registered with the local municipality was 6,090,244 in 2020, while the Japan Pet Food Association estimated that the number of dogs owned by households was 8,489,000 in the same year [31], and the annual rabies vaccination coverage was estimated to be approximately 50.4%. By contrast, MHLW officially reported a vaccination coverage of 70.2% in 2020. This coverage was also reported by the Japan Veterinary Medical Association [32].

This study had four main limitations. First, we were unable to determine the causal relationships between owner characteristics and dog vaccination and registration because the study was conducted using a cross-sectional survey with many unmeasured confounders, including the owners’ knowledge about rabies as a disease. Second, the survey was self-reported, and there may have been recall bias, especially regarding past rabies vaccinations. Third, the study participants were a convenient sample; thus, the findings may not be representative of the entire Japanese population. For this reason, we cannot apply our survey findings to the entire population of the country. Fourth, we did not investigate the owners’ detailed knowledge about rabies; therefore, the knowledge about rabies among the study participants may not have been sufficiently quantified.

## 5. Conclusions

Despite the study’s limitations, we believe that the findings strongly imply that improving dog owners’ knowledge about mandatory annual rabies vaccination and the opportunities for mass vaccination events may increase rabies vaccination coverage in dogs and will contribute to future rabies vaccination programs.

## Figures and Tables

**Table 1:** Characteristics of owners (*n* = 534) and dogs (*n* = 629), Japan.

Characteristics	Estimate
Owners’ age (years)	
Mean ± SD	45.7 ± 17.7 years
Range	15–79 years
Owners’ age group	
≤20 years	52 (9.7%) persons
21–40 years	170 (31.8%) persons
41–60 years	176 (33.0%) persons
≥61 years	136 (25.5%) persons
Gender (male)	284 (53.1%) persons
People residing in Western Japan	281 (52.6%) persons
The ratio of the number of dogs to household members	
Mean ± SD	0.49 ± 0.3
Dog age (years)	
Mean ± SD	7.3 ± 4.7 years
Range	0–21 years
Dog age group	
≤5 years	261 (41.5%) dogs
6–10 years	200 (31.8%) dogs
≥11 years	168 (26.7%) dogs
Rabies vaccination history (ever vaccinated)	610 (97.0%) dogs
Rabies vaccinations within 12 months	353 (56.1%) dogs
Registration of dogs	562 (89.3%) dogs
Vaccination other than rabies (ever vaccinated)	514 (81.7%) dogs

**Table 2:** Univariate associations between dog rabies vaccination and owner characteristics.

Section		Category (Category/Compared Group)	Numbers in the Corresponding Category	*p*-Value	Odds Ratio (95% CI)
Demographic characteristics	Education	University or Graduate school /Junior high school	317	0.014	2.5 (1.2–5.1)
Education associated with medicine	Knowing vaccination as mandatory by law	Yes/No	517	<0.001	2.9 (1.9–4.4)
	Knowing mass vaccination opportunity	Yes/No	453	<0.001	2.9 (2.0–4.1)
Veterinary service	Having a family veterinary clinic	Yes/No	551	<0.001	2.3 (1.4–3.7)
	Frequency of visiting veterinary clinics	Once a half year/ Once a year	312	0.011	2.0 (1.2–3.3)
		At least once a month/ Once a year	246	0.009	2.0 (1.2–3.5)
	Veterinarian’s advice on rabies vaccination	Yes/No	277	0.019	2.6 (1.2–5.7)

**Table 3:** Univariate associations between owner characteristics and non-rabies dog vaccinations.

Section		Category (Category/Compared Group)	Numbers in the Corresponding Category	*p*-Value	Odds Ratio (95% CI)
Demographic characteristics	Area	Urban/Rural	226	0.005	1.8 (1.2–2.9)
Education associated with medicine	Education history associated with medicine	Yes/No	131	0.005	2.3 (1.3–4.3)
	Having an occupation related with dogs	Yes/No	52	0.014	3.9 (1.2–12.9)
	COVID-19 vaccination history in owner	Yes/No	548	0.001	2.4 (1.5–4.1)
	Flu vaccination history in owner	Every year/Never	259	0.027	1.8 (1.1–3.0)
	Flu vaccination intension in owner	Yes/No	333	0.017	1.7 (1.1–2.5)
Veterinary service	Having a family veterinary clinic	Yes/No	551	<0.001	3.7 (2.2–6.1)
	Frequency of visiting veterinary clinics	Once a half year/Once a year	312	0.010	2.1 (1.2–3.8)
		At least once a month/ Once a year	246	0.005	2.4 (1.3–4.4)
Dog characteristics	Interaction with other dog owners	Once a week/No interaction	224	0.008	1.9 (1.2–3.0)
	Experience with the dog community	Yes/No	109	0.014	2.2 (1.2–4.3)
	Dog place	Indoor/Outdoor	585	<0.001	3.1 (1.6–6.0)
	Dog positioning	A member of the family/ A familiar animal	514	0.017	2.7 (1.2–4.3)

**Table 4:** Univariate associations between dog registration and owner characteristics.

Section		Category (Category/Compared Group)	Numbers in the Corresponding Category	*p*-Value	Odds Ratio (95% CI)
Demographic characteristics	Vaccination cost in dog	Not high/High	345	0.013	1.9 (1.2–3.2)
Education associated with medicine	Knowing vaccination as mandatory by law	Yes/No	517	<0.001	5.9 (3.5–10.1)
	Knowing mass vaccination opportunity	Yes/No	453	<0.001	3.2 (1.9–5.5)
	COVID-19 vaccination intension in owner	Yes/No	261	0.025	1.9 (1.1–3.3)
	Flu vaccination history in owner	Every year/Never	259	0.047	1.9 (1.0–3.7)
Veterinary service	Having a family veterinary clinic	Yes/No	551	<0.001	3.9 (2.1–7.2)
	Frequency of visiting veterinary clinics	Once a half year/ Once a year	312	<0.001	5.1 (2.7–9.5)
		At least once a month/Once a year	246	<0.001	6.4 (3.2–13.0)
	Veterinarian’s advice on rabies vaccination	Yes/No	277	0.004	2.4 (1.3–4.3)
Dog characteristics	Frequency of interaction with other dog owners	At least once a half year/No interaction	119	0.011	2.8 (1.3–6.1)
		Once a week/ No interaction	258	0.003	2.8 (1.6–5.1)
	Experience with a dog community	Yes/No	109	0.013	2.8 (1.1–7.2)

## Data Availability

All anonymized individual participant data reported in this paper are available online.

## Supplementary Materials

The following supporting information can be downloaded at: https://www.mdpi.com/article/10.3390/vaccines11020352/s1: Supplementary File S1: Preliminary questionnaire; Supplementary File S2: Questionnaire.
