# Supplementary material for: Epidemiological Associations between Rabies Vaccination and Dog Owner Characteristics
Source: Vaccines (Basel). 2023 Feb 3;11(2):352. doi: 10.3390/vaccines11020352 (PMC9962917; doi:10.3390/vaccines11020352)
Supplement: Supplementary file 1 [file vaccines-11-00352-s001.zip › vaccines-2198061-supplementary.pdf]

Supplementary File S1: Preliminary Questionnaire – English Version

Dear Participant,
Please ensure that you select only one of the options for each of the questions.

Question	Options
1. Do you own one or more dogs currently?<br>*Owning a dog means that you own one or more dogs where you live.	□ Own one or more currently<br>□Not currently, but have owned a dog previously<br>□Never owned a dog<br>□Unknown
2. How many dogs do you own at the moment?	□0<br>□1<br>□2<br>□3<br>□4<br>□5<br>□ Over 6
3. What type of residence do you live in?	□A private house<br>□A condominium<br>□Apartment<br>□Other ________________
4. What is your highest academic level?	□Primary school □Junior high school □High school<br>□University □Graduate school
5. How much is your yearly household income?<br>*Combined income before tax of all members living in the same household	□Less than 3 million yen<br>□Greater than 3 million yen, but less than 10 million yen<br>□Greater than 10 million yen

Supplementary File S2: Questionnaire – English Version

If you own more than five dogs, please answer for the five oldest.
Please select only one of the options for each question.
For the second to fourth and seventh to ninth questions, please tick the option that applies to each dog.

<table>
<tr><td>Question</td><td>Options</td></tr>
<tr><td>1. How many dogs do you currently own?</td><td>☐0
☐1
☐2
☐3
☐4
☐5
☐ Over 6</td></tr>
<tr><td>2. How did you obtain your dog?</td><td>
<table>
<tr><td></td><td>1
(1st
oldest
dog)</td><td>2</td><td>3</td><td>4</td><td>5
(5th
oldest
dog)</td></tr>
<tr><td>Bought</td><td>☐</td><td>☐</td><td>☐</td><td>☐</td><td>☐</td></tr>
<tr><td>Was given</td><td>☐</td><td>☐</td><td>☐</td><td>☐</td><td>☐</td></tr>
<tr><td>Offspring of a dog that I already owned.</td><td>☐</td><td>☐</td><td>☐</td><td>☐</td><td>☐</td></tr>
<tr><td>Took in as a stray</td><td>☐</td><td>☐</td><td>☐</td><td>☐</td><td>☐</td></tr>
<tr><td>Other</td><td>☐</td><td>☐</td><td>☐</td><td>☐</td><td>☐</td></tr>
<tr><td>Not applicable (e.g. because I don’t have 2 or more dogs)</td><td>☐</td><td>☐</td><td>☐</td><td>☐</td><td>☐</td></tr>
</table>
</td></tr>
<tr><td>3. Is your dog local or exotic?</td><td>
<table>
<tr><td></td><td>1
(1st
oldest
dog)</td><td>2</td><td>3</td><td>4</td><td>5
(5th
oldest
dog)</td></tr>
<tr><td>Local (with pedigree)</td><td>☐</td><td>☐</td><td>☐</td><td>☐</td><td>☐</td></tr>
</table>
</td></tr>
</table>

Exotic (with pedigree)	☐	☐	☐	☐	☐
Local (no pedigree)	☐	☐	☐	☐	☐
Exotic (no pedigree)	☐	☐	☐	☐	☐
Hybrid	☐	☐	☐	☐	☐
Unknown	☐	☐	☐	☐	☐

4. Where do you keep your dog?

	1 (1st oldest dog)	2	3	4	5 (5th oldest dog)
Indoors	☐	☐	☐	☐	☐
Outdoors (On a leash)	☐	☐	☐	☐	☐
Outdoors (No leash)	☐	☐	☐	☐	☐
No applicable dog	☐	☐	☐	☐	☐

5. How old was your dog when you first obtained it?

1: ________________
2: ________________
3: ________________
4: ________________
5: ________________

6. How old is your dog now?

1: ________________
2: ________________
3: ________________
4: ________________
5: ________________

7. Please check all ages at which your dog was vaccinated against rabies.

	1 (1st	2	3	4	5 (5th

	oldest dog)				oldest dog)
1 year old	□	□	□	□	□
2	□	□	□	□	□
3	□	□	□	□	□
4	□	□	□	□	□
5	□	□	□	□	□
6	□	□	□	□	□
7	□	□	□	□	□
8	□	□	□	□	□
9	□	□	□	□	□
10	□	□	□	□	□
11	□	□	□	□	□
12	□	□	□	□	□
13	□	□	□	□	□
14	□	□	□	□	□
15	□	□	□	□	□
16	□	□	□	□	□
17	□	□	□	□	□
18	□	□	□	□	□
19	□	□	□	□	□
20	□	□	□	□	□
21	□	□	□	□	□
22	□	□	□	□	□
23	□	□	□	□	□
24	□	□	□	□	□
25	□	□	□	□	□
Never	□	□	□	□	□

No applicable dog	☐	☐	☐	☐	☐

8. Did you register your dog?

	1 (1st oldest dog)	2	3	4	5 (5th oldest dog)
Registered at the same time as a rabies vaccination	☐	☐	☐	☐	☐
Only rabies vaccination	☐	☐	☐	☐	☐
Only registration	☐	☐	☐	☐	☐
Not vaccinated or registered	☐	☐	☐	☐	☐
No applicable dog	☐	☐	☐	☐	☐

9. Have you ever vaccinated your dog for a disease other than rabies?

	1 (1st oldest dog)	2	3	4	5 (5th oldest dog)
Yes	☐	☐	☐	☐	☐
No	☐	☐	☐	☐	☐
No applicable dog	☐	☐	☐	☐	☐

10. How many people are there in your household? (Please don't include your pets; only humans)

☐1
☐2
☐3
☐4
☐5
☐6
☐______

11. Where do you live?
*Urban areas are considered to be the 23 wards of Tokyo and ordinance-designated cities (where "ward" is

☐Urban
☐Rural

included in the address).	
12. Have you received any education related to medicine or animal health (i.e., underwent education about pathogens, pathology, and/or treatment of diseases in humans or animals)?	□Yes<br>□No
13. Do you have a full-time position/occupation related to dogs (i.e., at a pet shop, as a breeder, or veterinarian)?	□Yes<br>□No
14. Do you have a full-time position/occupation associated with medicine or animal health (i.e., doctor, pharmacist, nurse, or veterinarian)?	□Yes<br>□No
15. Have you ever been vaccinated against COVID-19?	□Yes<br>□No
16. Would you still want to receive a COVID-19 vaccine if there was a charge for it?	□Yes<br>□No
17. How often do you get vaccinated against influenza?	□Every year<br>□Every few years<br>□Never
18. Do you intend to get an influenza vaccination in the future?	□Yes<br>□No
19. How do you think about the dogs that you own?	□As a member of the family<br>□As a familiar animal-like family<br>□As a familiar animal
20. Do you interact with other dog owners in	□Yes<br>□No

your neighborhood (i.e., talking to other dog owners when walking your dog)?	
21. How often do you interact with other dog owners in your neighborhood? (Only for those who answered Yes to Q20)	□Once a week<br>□Once a month<br>□Once every six months to a year<br>□Once every several years
22. Have you ever joined a community of dog lovers?	□Yes<br>□No
23. Do you have a family veterinary clinic where you consult about your dog's health?	□Yes<br>□No
24. How often do you visit a veterinary clinic?	□Once a week<br>□Once a month<br>□Once every six months to a year<br>□Once every several years
25. Has a veterinarian ever advised you about vaccinating your dog against rabies?	□Recommended vaccinating your dog against rabies<br>□Recommended not vaccinating your dog against rabies<br>□No experience
26. Are you aware that dog rabies vaccination is compulsory every year?	□Yes<br>□No
27. Are you aware that there is an annual mass rabies vaccination opportunity from April to June organized by the local municipality?	□Yes<br>□No
28. Do you feel that rabies vaccination is expensive?	□Expensive<br>□Not expensive
